# Experimental Study on Blue Light Interaction with Human Keloid-Derived Fibroblasts

**DOI:** 10.3390/biomedicines8120573

**Published:** 2020-12-06

**Authors:** Giada Magni, Martina Banchelli, Federica Cherchi, Elisabetta Coppi, Marco Fraccalvieri, Michele Rossi, Francesca Tatini, Anna Maria Pugliese, Duccio Rossi Degl’Innocenti, Domenico Alfieri, Paolo Matteini, Roberto Pini, Francesco S. Pavone, Francesca Rossi

**Affiliations:** 1 Istituto di Fisica Applicata “Nello Carrara”, Consiglio Nazionale delle Ricerche (CNR-IFAC), 50019 Florence, Italy; m.banchelli@ifac.cnr.it (M.B.); michele.rossi.od@gmail.com (M.R.); f.tatini@ifac.cnr.it (F.T.); r.pini@ifac.cnr.it (R.P.); f.rossi@ifac.cnr.it (F.R.); 2 Department of Neuroscience, Psychology, Drug Research and Child Health, Section of Pharmacology and Toxicology, University of Florence, 50139 Florence, Italy; federica.cherchi@unifi.it (F.C.); elisabetta.coppi@unifi.it (E.C.); 3 SCDU Chirurgia Plastica, ASO Città della Salute e della Scienza di Torino, 10133 Turin, Italy; marco@fraccalvieri.it; 4 EmoLED s.r.l., 50019, Florence, Italy; d.rossi@emoled.com (D.R.D.); rs@emoled.com (D.A.); 5 Department of Physics, University of Florence, 50019 Florence, Italy; francesco.pavone@unifi.it; 6 European Laboratory for NonLinear Spectroscopy (LENS), 50019 Florence, Italy; 7 Istituto Nazionale di Ottica, Consiglio Nazionale delle Ricerche (CNR-INO), 50125 Florence, Italy

**Keywords:** blue light, LED, photobiomodulation, skin fibrosis, keloid, fibroblast, Cytochrome C, Raman spectroscopy, patch-clamp

## Abstract

Keloids are an exuberant response to wound healing, characterized by an exaggerated synthesis of collagen, probably due to the increase of fibroblasts activity and to the reduction of their apoptosis rate: currently no standard treatments or pharmacological therapies are able to prevent keloid recurrence. To reach this goal, in recent years some physical treatments have been proposed, and among them the PhotoBioModulation therapy (PBM). This work analyses the effects of a blue LED light irradiation (410–430 nm, 0.69 W/cm^2^ power density) on human fibroblasts, isolated from both keloids and perilesional tissues. Different light doses (3.43–6.87–13.7–20.6–30.9 and 41.2 J/cm^2^) were tested. Biochemical assays and specific staining were used to assess cell metabolism, proliferation and viability. Micro-Raman spectroscopy was used to explore direct effects of the blue LED light on the Cytochrome C (Cyt C) oxidase. We also investigated the effects of the irradiation on ionic membrane currents by patch-clamp recordings. Our results showed that the blue LED light can modulate cell metabolism and proliferation, with a dose-dependent behavior and that these effects persist at least till 48 h after treatment. Furthermore, we demonstrated that the highest fluence value can reduce cell viability 24 h after irradiation in keloid-derived fibroblasts, while the same effect is observed 48 h after treatment in perilesional fibroblasts. Electrophysiological recordings showed that the medium dose (20.6 J/cm^2^) of blue LED light induces an enhancement of voltage-dependent outward currents elicited by a depolarizing ramp protocol. Overall, these data demonstrate the potentials that PBM shows as an innovative and minimally-invasive approach in the management of hypertrophic scars and keloids, in association with current treatments.

## 1. Introduction

The word “keloid” appeared for the first time in 1817, when Alibert used this term to describe the lesions as cancroid, or “crab claw-like appearance” (as the original meaning in ancient Greek) lesions, to define the lateral expansion of an excessive scar into the surrounding healthy tissue [1]. Years later, Mancini (in 1962) and Peacock (in 1970) introduced different definitions to identify keloids and hypertrophic scars [2]. Nowadays, keloids are clinically described as human fibroproliferative benign dermal tumors characterized by an excessive synthesis and accumulation both of collagen and extracellular matrix (ECM) elements [3]. For these reasons, keloids can extend beyond the edge of the original wound, they do not spontaneously regress and increase their dimensions steadily. These features impact on the differential diagnosis, in which the keloid is distinguished from the hypertrophic scar [4]. Despite the research efforts to understand this skin fibrosis in recent years, both its etiology and pathogenesis have been poorly elucidated. In most cases, keloid scars represent an abnormal outcome of a wound healing process, triggered by surgery, insect bites, piercing, tattoo, burns, trauma, injuries, and acne. Spontaneous keloids, which occur without any stimuli, are rare [5]. Keloids exhibit peculiar features: the dimensions are in the range from few millimeters to several centimeters, also depending on the site of the lesion; their surface is smooth and shiny, with different thickness, and a compactness ranging from mildly tender to firm; the borders are delineated, but irregular in outline; the color can vary from purple to pink and generally it is always highly pigmented in respect to the healthy surrounding skin [6]. This kind of skin fibrosis is common in subjects which exhibit a high Fitzpatrick’s index associated with a family history of keloid occurrence. The main symptoms concern itching, pain and difficulty in movements in severe cases, but also the psychological sphere and the quality of life can be affected [7,8,9]. Although several pharmacological and not-pharmacological therapies exist and the increasing number of novel strategies is emerging, the prevention of the keloid recurrence remains a clinical challenge [10]. Moreover, keloids occur only in humans and the lack of animal models increases the efforts to study this pathology by in vitro analysis, in particular in keloid-derived cultured cells [11]. Recently, PhotoBioModulation therapy (PBM) has gained importance as a physical therapy in the treatment of wounds, burns etc. [12], evidencing the possibility of modulating cell activity and cell proliferation with a biphasic dose behavior [13]. In some preliminary studies [14,15] we showed that the use of blue light can induce a modulation in fibroblasts activity, in particular in those cells extracted from skin samples of patients suffering from keloid recurrence. In the present study we investigate the effects of different doses of blue LED light (410–430 nm) on fibroblasts isolated from keloids and perilesional tissues, at different irradiation doses, to point out whether PBM with blue light can be applied in keloid scars to prevent their reappearance.

## 2. Materials and Methods

### 2.1. The Blue LED Light Device and Its Application on Human Cultured Fibroblasts

The blue LED device utilized in these experiments is based on commercially available LED, emitting at around 420 nm with 1 W (Watt) optical emission power. The device is fiber-coupled: it consists in a bench top device furnished with a flexible polymeric fiber, 1.2 m in length, equipped with a touch screen where it is possible to control all the irradiation parameters, such as irradiation time and power. The illuminated area corresponded to a circle of 6.4 mm in radius. The resulting power density was 0.69 W/cm^2^. The intensity distribution of the light at the tissue surface was homogeneous, as from a top hat intensity light source. The fluence values were calculated taking into account the dimension of the plate or well and the irradiation spot, at the maximum energy provided by the device. All the irradiation parameters were measured using a photodiode energy sensor (Ophir, Darmstadt, Germany). The fluence values which correspond to different doses of blue light directly applied to cultured cells, are reported in Table 1.

### 2.2. Human Keloid Samples

**Figure 1:**
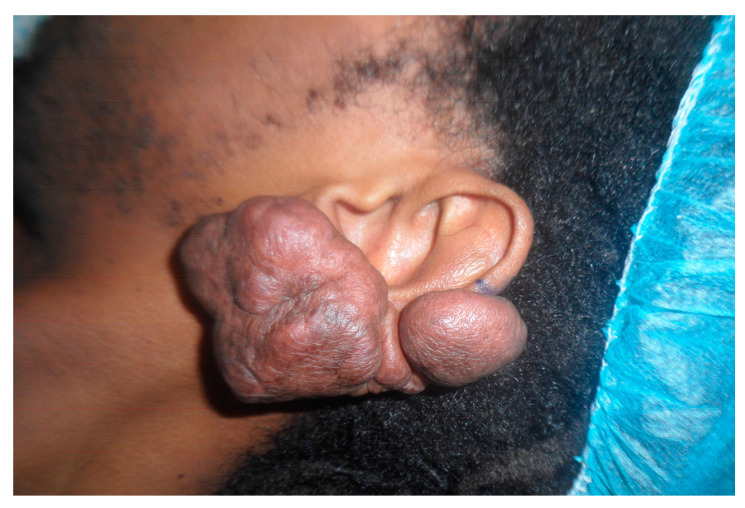
Example of human keloid tissue.

All subjects provided written informed consent approved by the Ethical Committee of AOU Città della Salute e della Scienza di Torino, A.O. Ordine Mauriziano di Torino- A.S.L. Città di Torino, Turin, Italy (Prot. N° 0073787, 25/07/2017). All the experiments were performed in accordance with the Helsinki declaration and Good Clinical Practice in the matter of ethical principles and privacy of the human subjects. The differential diagnosis from hypertrophic scars and the score assigned in Fitzpatrick scale were assessed by the aesthetic surgeon. Eleven keloid samples (Figure 1) were obtained from eleven patients (nine males and two females, average age 32.8±10.32 SD) subjected to reconstructive and aesthetic surgeries. From eight out of eleven patients, it was possible to obtain keloid perilesional tissue. In eight patients, the keloid tissue was already removed during previous manual surgeries (five patients) or laser-assisted surgeries (three patients). The general and clinical characteristics of all excised-keloid tissues are depicted in Table 2. Informed consent was obtained from the patient.

### 2.3. Cell Cultures

Immediately after biopsy, both keloid and perilesional tissues were collected in Dulbecco Modified Eagle Medium (DMEM, PanReact AppliChem GmbH, Darmstadt, Germany), maintained at 4 °C and used within 5 h from the explant. Each sample was subjected at least to 6 vigorous washes in phosphate-buffered saline (PBS, PanReact AppliChem GmbH, Darmstadt, Germany) to remove hair and blood residues. After that, keloids and perilesional tissues were dissected with scalpels or scissors to obtain sections of about 3 mm in diameter. The fragments were disposed on scratched-Petri dishes keeping semi-opened under laminar flow for about 40 min to allow the adhesion of each sample to the plate [16]. After this procedure, low glucose DMEM supplemented with 10% fetal bovine serum (FBS), 1% of glutamine and 1% of penicillin–streptomycin (both purchased from EuroClone S.p.A., Milan, Italy) was added and the cells were kept at 37 °C and 5% CO_2_. Fibroblasts migrated from the tissue to the bottom of the dish and within three weeks from the preparation of the cultures, fibroblasts were detached with Trypsin-EDTA 0.25% solution (Sigma-Aldrich, Milan, Italy), collected in a centrifuge tube and centrifuged at 1000 rpm for 6 min (Thermo CL10, Milan, Italy); the pellet was then seeded in T25 flask (Greiner Bio-One Italia, Milan, Italy). The cells were maintained under standard culture conditions (37 °C and 5% CO_2_) and medium refreshed every 48 h. Cells were splitted in T75 flask (Greiner Bio-One Italia, Milan, Italy) when reaching about 80% of confluence.

### 2.4. Immunocytochemical Staining

Cells were seeded in 35 mm^2^ imaging dishes with a polymer coverslip bottom (Ibidi GmbH, Martinsried, Germany) and immunocytochemical staining was performed as follows. Cells were fixed using 3.6% paraformaldehyde (PFA, Sigma-Aldrich, Milan, Italy) in 0.1 M and 7.4 pH PBS solution for 5 min at room temperature (RT). After that, 2 washes with PBS were performed to remove the excess of fixative. Permeabilization was executed using 0.25% Triton X100 (Sigma-Aldrich, Milan, Italy) in PBS for 10 min on a mechanical shaker at RT. The unspecific sites were blocked by incubation in 1% of BSA (bovine serum albumin) or 10% of goat serum (both purchased from Sigma-Aldrich, Milan, Italy) in PBST (PBS with 0.1% of Tween20, Sigma-Aldrich, Milan, Italy). Anti-Heat Shock Protein-47 was diluted 1:250, while anti-type I collagen and *α*SMA antibodies were diluted 1:500 in PSBT solution. All secondary antibodies, AlexaFluor555, AlexaFluor488 and AlexaFluor647, were diluted 1:600 in PBST solution. All antibodies were purchased from AbCam (Cambridge, UK) and used accordingly to the manufacturer instructions. DAPI (4′,6-diamidino-2-phenylindole, Sigma-Aldrich, Milan, Italy) was added to stain cell nuclei and to mount the coverslip. Immunocytochemical images were obtained by SP8 laser scanning confocal microscope (Leica Microsystems, Mannheim, Germany), using a 40× water-immersion objective (NA 1.40 Plan). The collected images were analyzed with an open-source software (ImageJ, version 1.49v National Institutes of Health, Bethesda, MD, USA). Control experiments were performed by incubating fixed cells only with the secondary antibodies and DAPI to exclude nonspecific binding.

### 2.5. CCK-8 Assay

Cellular metabolic activity was measured by Cell Counting Kit-8 (CCK-8) assay (Sigma-Aldrich, Milan, Italy). The CCK-8 uses WST-8, which produces a water-soluble formazan dye according to the dehydrogenase activity in the presence of 1-methoxyphenazine methosulfate, a stable electron carrier mediator between NAD(P)H and tetrazolium dyes. Colorless WST-8 is bioreduced by cellular dehydrogenases and becomes WST-8 formazan with an orange color that is soluble in the tissue culture medium [17,18]. CCK-8 assay was used by following the manufacturer’s instructions. Fibroblasts cells were counted using Neubauer chamber (Karl Hecht Assistent GmbH, Sondheim vor der Rhön, Germany), 5 × 10^3^ cells were seeded in 96-multiwell plates (Greiner Bio-One Italia, Milan, Italy) and maintained for 24 h in standard culture conditions (37 °C and 5% CO_2_) before the experiments. To avoid interferences, such as double or partial irradiation, cells were seeded in alternate wells, in rows and columns, for each multiwell. The day after, DMEM was replaced with SFM DMEM and the cells were irradiated by the blue LED light applying the following doses in 18 wells: 3.43–6.87–13.7–20.6–30.9 and 41.2 J/cm^2^. For each experiment, three wells were left untreated and used as control while other three wells were used to measure the background of multiwell. The irradiation was performed by holding the fibre steadily 12 mm far from the bottom of each well. The absorbance at 450 nm (reference wavelength at 630 nm) was read using an automatic microplate absorbance reader (LT-4000 Labtech, Heathfield, East Sussex, England). Each experiment was performed at least in triplicate.

### 2.6. Sulforhodamine B Assay

Cell proliferation was evaluated by Sulforhodamine B based (SRB) assay purchased from Sigma-Aldrich (Milan, Italy). Sulforhodamine binds stoichiometrically to proteins under mildly acidic conditions and then can be extracted under basic conditions; thus, the amount of bound dye can be used as an approximation of cell mass, which can then be extrapolated to measure cell proliferation [19,20,21]. The human keloid fibroblast cells were counted, maintained, and treated with the blue LED light using the same protocol as CCK-8, described in 2.5. The absorbance at 570 nm was read using an automatic microplate absorbance reader (LT-4000 Labtech, Heathfield, East Sussex, England). The experiments were performed at least in triplicate.

### 2.7. Viability Assay

Cell viability was assessed by using Trypan Blue solution 0.4% (Sigma-Aldrich, Milan, Italy) which is not absorbed by healthy and viable cells, but that stains cells with a damaged membrane, in blue or light blue color. Fibroblast cells were seeded in 35 mm^2^ dishes (Greiner Bio-One Italia, Milan, Italy) and were maintained in low glucose DMEM for 24 h. Treated samples were irradiated with the blue LED light applying 41.2 J/cm^2^, whereas control samples were kept not irradiated. The treatment was performed in serum-free medium (SFM DMEM) without red phenol, to avoid possible interferences during the irradiation. All the dishes were divided into 2 groups and were evaluated 24 or 48 h after irradiation; during this time cells were maintained under standard culture conditions (37 °C and 5% CO_2_). After the removal of the SFM DMEM, 2 washes in PBS were performed and Trypan Blue solution diluted 1:4 in PBS was applied for 6 min to all the dishes. After 2 additional washes in PBS, cells were immediately observed under an inverted optical microscope (Eurotek Orma, INV100T, Milan, Italy) using 10X magnification. Ten random images for each sample were acquired using a 5-megapixel photo-camera (Eurotek Orma, Milan, Italy) and Manta software (MANTA, New York, NY, USA) was used to analyze the collected images. Two separate and blind counts were carried out by trained personnel. The experiment was performed at least in triplicate.

### 2.8. Electrophysiological Recordings

Whole-cell patch-clamp recordings were performed in −60 mV clamped-cells as described [14,22]. The solutions were used as follows; extracellular solution (mM): NaCl 147; KCl 4; MgCl_2_ 1; CaCl_2_ 2; HEPES (4-(2-hydroxyethyl)-1-piperazine ethanesulfonic acid) 10; D-glucose 10 (pH 7.4 with NaOH). Standard K^+^-based pipette solution (mM): K-gluconate 130; NaCl 4.8; KCl 10; MgCl_2_ 2; CaCl_2_ 1; Na_2_-ATP 2; Na_2_-GTP 0.3; EGTA 3; HEPES 10 (pH 7.4 with KOH). Cells were plated into 13 mm diameter coverslips and allowed to adhere before starting the recordings. Each sample was transferred to a 1 mL recording chamber mounted on the platform of an inverted microscope (Olympus CKX41, Milan, Italy) superfused at a flow rate of 1.5 mL/min by a three-way perfusion valve controller (Harvard Apparatus, Holliston, MA, USA). Borosilicate glass electrodes (Harvard Apparatus, Holliston, MA, USA) were pulled with a Sutter Instruments puller (model P-87) to a final tip resistance of 3–5 MΩ. Data were acquired with an Axopatch 200B amplifier (Axon Instruments, Union City, CA, USA), low-pass filtered at 10 kHz, stored and analyzed with pClamp 9.2 software (Axon Instruments, Union City, CA, USA). A voltage ramp protocol (800 ms depolarization from −80 to +80 mV) was repeated every 15 s to evoke overall voltage-dependent currents before, during, and after the application of 20.6 J/cm^2^ of blue LED light. Total outward currents evoked by the voltage ramp were quantified by measuring current amplitude at +80 mV. Control values were obtained by averaging the last 4 traces (1 min) of baseline and were compared to those measured during the fifth minute after irradiation. Net blue LED light-activated currents, or blue LED light-sensitive currents, were obtained in each cell by subtraction of the control ramp from the ramp recorded at minute five after treatment. In averaged results, current amplitude (in pA) was normalized to cell capacitance (in pF) and expressed as pA/pF.

### 2.9. Raman Spectroscopy Measurements

Raman spectroscopy was used to investigate the redox states and changes in photosensitive species, in particular on Cytochrome C (Cyt C) oxidase, an important player involved in the mitochondrial transport chain and considered one of the main mediator of the cell response to light stimuli [23]. Raman experiments were previously described [14]. Briefly, 2 µL of the fibroblasts pellet was drop-casted onto a gold mirror support (ME1S-M01; Thorlabs, Inc., Newton, NJ, USA) and Raman spectra were immediately recorded on not irradiated samples (n = 2) and after the application of 20.6 J/cm^2^ (n = 2) or 41.2 J/cm^2^ (n = 2) of blue LED light. For each sample at least 20 individual cells were inspected while carefully avoiding cell dehydration. By working at low power laser intensities, we did not observe any possible photoreduction or photooxidation of Cyt C. As a reference of the data obtained from measurements on keloid or perilesional fibroblasts, also 200 mM solution of Cyt C was subjected to the same doses of blue light. A total of 15 Raman spectra were recorded on the Cyt C samples before the blue LED light irradiation and after the application of 20.6 J/cm^2^ (n = 2) and 41.2 J/cm^2^ of blue light treatment. All data were baseline corrected when needed.

### 2.10. Statistical Analysis

Data obtained from CCK-8, SRB assays and electrophysiological recordings were expressed as mean ± SEM (Standard Error of the Mean). Student’s paired or unpaired *t*-tests and one-way ANOVA followed by Dunnett’s multiple comparison test analysis were performed. All data were analyzed by using commercial software package GraphPad Prism (GraphPad Software, San Diego, CA, USA). Data obtained from cell viability evaluation were expressed as mean ± SD (Standard Deviation). The analysis of cells viability experiments was performed as follows: the hypothesis of normality was verified using the Kolmogorov-Smirnov test. This allowed us to evaluate the most suitable type of statistical analysis. This led to the choice of the two-sample two-tailed *t*-test to verify the equality of the treated/control means separately at 24 and 48 h. A variance analysis of repeated measures was then performed for a multiple comparison between 24 and 48 h. Through the Bartlett test it was found that the assumption of homoskedasticity in the variables is not respected and this led us to choose a non-parametric approach by opting for the Friedman test. This analysis was performed using Rcommander open-source software (https://www.rcommander.com/; version: 2.7-1, Vienna, Austria). Statistical significance was set at * *p* < 0.05 for all the experimental results.

## 3. Results

### 3.1. Characterization of Cell Cultures Isolated from Keloid and Perilesional Tissues by Immunocytochemical Staining

**Figure 2:**
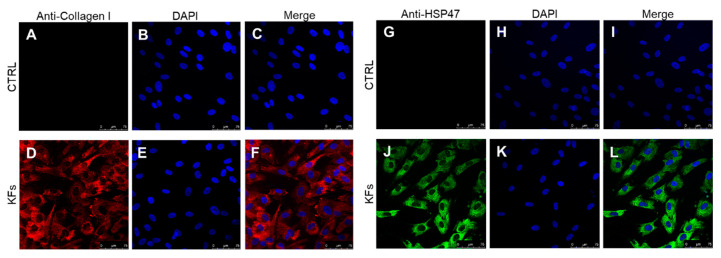
(**A**–**L**) Cultured keloid-derived fibroblasts (KFs) characterized by confocal microscopy. The use of selective markers such as type I collagen (in red) (**D**) and HSP47 (in green) (**J**) reveals the presence of fibroblast cells in primary cultures. All the visible nuclei (in blue) (**E**,**K**) co-localize with the type I collagen (in red) (**F**) or with HSP47 (in green) (**L**) in KFs. Control experiments were performed in both cases to verify type I collagen and HSP47 antibodies selectivity (**C**,**I**), respectively. Secondary antibodies were used without primary antibodies (**A**,**G**) and cultured cells were stained by DAPI (**B**,**H**). Scale bar: 75 µm.

**Figure 3:**
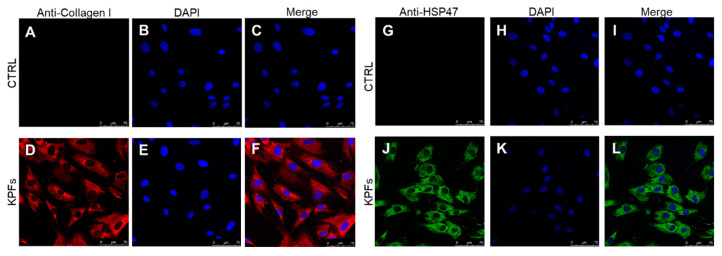
(**A**–**L**) Cultured fibroblasts isolated from perilesional tissues (KPFs) characterized by confocal microscopy. The use of selective markers such as type I collagen (in red) (**D**) and HSP47 (in green) (**J**) reveals the presence of fibroblast cells in primary cultures. All the visible nuclei (colored in blue) (**E**,**K**) co-localize with the type I collagen (in red) (**F**) or with HSP47 (in green) (**L**) in KPFs. Control experiments were performed in both cases to verify type I collagen and HSP47 antibodies selectivity (**C**,**I**), respectively. Secondary antibodies were used without primary antibodies (**A**,**G**) and cultured cells were stained by DAPI (**B**,**H**). Scale bar: 75 µm.

**Figure 4:**
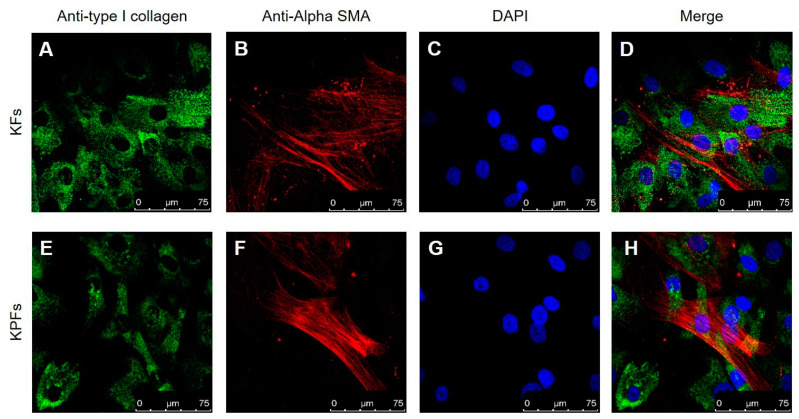
(**A**–**H**) Myofibroblasts characterization by confocal microscopy in keloid fibroblast cells (KFs) and perilesional fibroblast cells (KPFs). A selective antibody for actin was used and a small population of myofibroblasts was identified both in KFs and in KPFs. (**A**,**E**) Expression of type I collagen (in green) in KFs and in KPFs, respectively. (**B**,**F**) Expression of *α*-SMA (in red) in KFs and in KPFs, respectively. (**C**,**G**) Cellular nuclei (in blue) labelled with DAPI in KFs and in KPFs, respectively. (**D**,**H**) Merge of (**A**,**B**,**C**) in KFs and (**E**,**F**,**G**) in KPFs. Scale bar: 75 µm.

Cultured skin fibroblasts isolated from keloids and perilesional tissues were characterized using immunocytochemical staining, as assessed by confocal microscopy. It is well established that fibroblast cells can synthesize different types of collagen, not only in both physiological and in pathological conditions, but also in vitro [24,25]. In our experiments we labelled cytosolic type I collagen and Heat Shock Protein-47 (HSP47), a chaperone molecule involved in the folding of different types of protein, among which type I collagen [26]; for this reason, HSP47 is considered a cytosolic precursor of collagen molecule [27,28]. As shown in the following figures, we found a similarly cytosolic signal of type I collagen-positive cells and HSP47-positive cells both in fibroblasts isolated from keloid tissue (Figure 2D,J) and from perilesional tissue (Figure 3D,J). Furthermore, these signals are co-localized with the total cell nuclei, demonstrating the presence of fibroblasts cells in the cultures obtained from keloids tissues (Figure 2F,L) and from perilesional tissues (Figure 3F,L). Moreover, it is known that myofibroblasts are typically found in fibrotic skin tissues such as keloids [29,30]. Thus, we evaluated the presence of myofibroblasts, revealed by the anti-alpha-smooth muscle actin (*α*-SMA) antibody, in cell cultures isolated from keloid tissue or perilesional tissue. We found type I collagen and *α*-SMA positive cells, identifying them as myofibroblasts. We did not quantify these cells, but they are definitely fewer compared to *α*-SMA negative cells (Figure 4D,H).

### 3.2. Blue LED Light Modulates Cell Metabolism in a Dose-Dependent Manner

**Figure 5:**
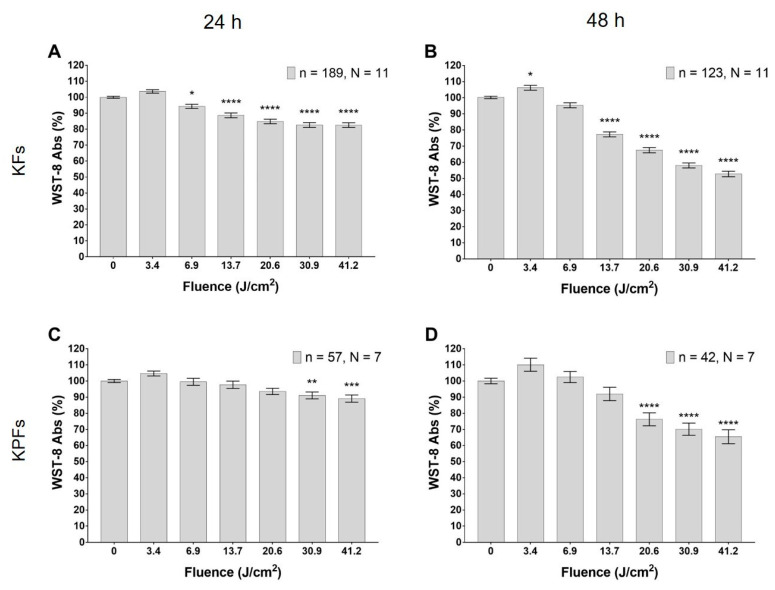
Effects of blue LED light on cell metabolism in human keloid fibroblasts. (**A**,**B**) Metabolism 24 and 48 h after the treatment in keloid fibroblasts, respectively. (**C**,**D**) Metabolism 24 and 48 h after the treatment in perilesional keloid fibroblasts, respectively. Data are expressed as mean ± SEM. Each measure is repeated in triplicate at 24 h and in duplicate at 48 h after treatment. Statistical analysis: * *p* < 0.05; ** *p* < 0.01; *** *p* < 0.001; **** *p* < 0.0001 vs. control (not irradiated cells), one-way ANOVA followed by Dunnett’s multiple comparison test.

The treatment with the blue LED light induces a significant decrease in keloid fibroblasts metabolism, measured with WST-8 (tetrazolium salt) method. This effect is dose-dependent, it begins 24 h from the irradiation (Figure 5A) and becomes more pronounced after 48 h (Figure 5B). In particular, 24 h after the treatment, the decrease reaches significant levels at fluence values ranging from 6.87 to 41.2 J/cm^2^ (Figure 5A); while at 48 h, a significant decrease is reached at fluences from 13.6 to 41.2 J/cm^2^ (Figure 5B). Noteworthily, the lowest dose tested (3.43 J/cm^2^) causes a significant increase in fibroblast metabolism 48 h after irradiation (Figure 5B). A significant decrease in cell metabolism is observed also in fibroblasts isolated from keloid perilesional tissues, both 24 and 48 h after blue LED light irradiation (Figure 5C,D). In detail, Figure 5C shows a significant reduction in cell metabolism at fluences ranging from 30.9 to 41.2 J/cm^2^, while after 48 h, even lower doses are effective in reducing cell metabolism (in the range 20.6–41.2 J/cm^2^; Figure 5D). The behavior at higher fluence has been investigated also with the trypan blue exclusion test (see Section 3.4).

### 3.3. Blue LED Light Modulates Cell Proliferation in a Dose-Dependent Manner

**Figure 6:**
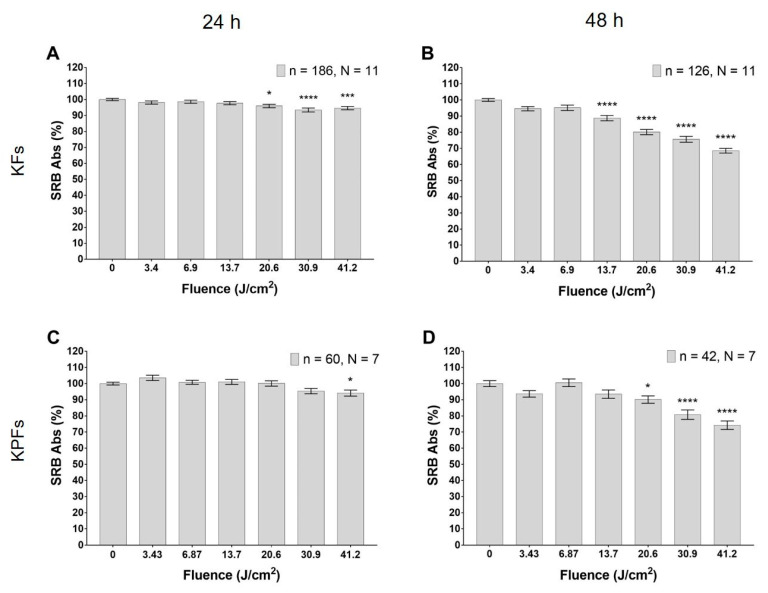
Effects of blue LED light on cell proliferation in human keloid fibroblasts. (**A**,**B**) Proliferation observed 24 and 48 h after the treatment in keloid fibroblasts, respectively. (**C**,**D**) Proliferation observed 24 and 48 h after the treatment in perilesional keloid fibroblasts, respectively. Data are expressed as mean ± SEM. Each measure is repeated in triplicate at 24 h and in duplicate at 48 h. Statistical analysis: * *p* < 0.05; *** *p* < 0.001; **** *p* < 0.0001 vs. control (not irradiated cells), one-way ANOVA followed by Dunnett’s multiple comparison test.

Cultured fibroblasts isolated from keloid tissues reduce their proliferation rate with an application of the blue LED light fluence in the range from 20.6 to 41.2 J/cm^2^ (Figure 6A), as observed 24 h after treatment. This effect is even more pronounced 48 h after treatment, when the sulforhodamine B (SRB) absorbance decreases in correspondence to fluence values ranging from 13.9 to 41.2 J/cm^2^ (Figure 6B). The reduction of fibroblasts proliferation by the application of the blue LED light occurs in a strictly dose-dependent manner. In fibroblasts isolated from keloid perilesional tissues, only the higher dose of irradiation with blue LED light induces a decrease in SRB absorbance after 24 h (Figure 6C), while at 48 h, the doses of 20.6, 30.9 and 41.2 J/cm^2^ can significantly reduce SRB absorbance (Figure 6D).

### 3.4. High Dose of Blue Led Light Reduces Cells Viability in Fibroblasts Isolated from Keloids and Perilesional Tissues

To confirm the results obtained with the tests carried out on metabolism and proliferation, we performed a cell viability assay by testing the highest dose of blue LED light. The dye exclusion test was used to determine the number of total viable cells counted in the treated and in the control samples after 24 and 48 h. After the staining, we found no stained cells in any sample. However, we observed that the number of cells in some samples was visibly lower than in others. For this reason, 10 random images were captured from each sample and cells were counted. Data from controls and treated samples were compared and can be viewed in Table 3. Our results show that the application of 41.2 J/cm^2^ significantly reduces the number of total keloid fibroblasts, 48 h after the treatment with the blue LED light, while at 24 h from the treatment, no significant differences are found. The same experimental procedures were also performed in fibroblasts isolated from perilesional tissue. In this case, our results showed that after 24 h, a 41.2 J/cm^2^ fluence induces a decrease in the total number of fibroblasts cell, in comparison to the control sample. This result demonstrates that the reduction observed by applying the fluence of 41.2 J/cm^2^ in WST-8 and SRB assays, should be ascribed to a cytotoxic effect.

### 3.5. Blue LED Light Increases the Outward Currents in Fibroblasts Isolated from Keloid Tissues

**Figure 7:**
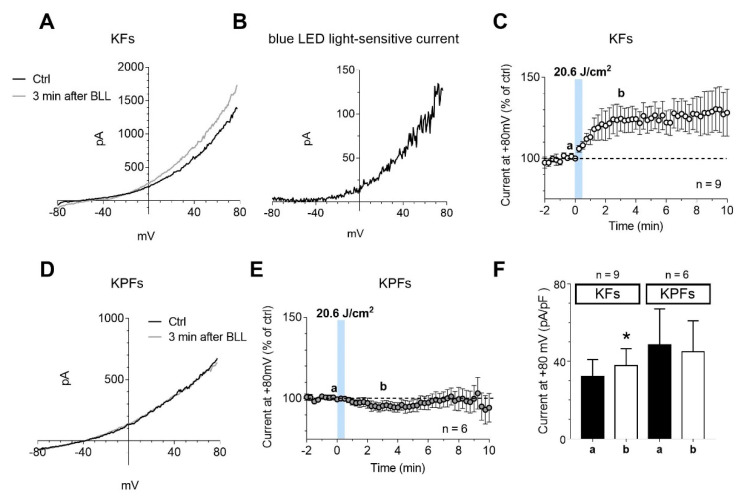
The application of blue LED light increases outward currents only in cultured keloid fibroblasts. (**A**,**D**) Original whole-cell patch clamp current traces evoked by a voltage ramp protocol (from −80 to +80 mV, 800 ms) before (Ctrl, black traces) or 3 min after the application of 20.6 J/cm^2^ of blue LED light (BLL, grey traces) in a typical keloid fibroblast (KF, A) or perilesional keloid fibroblast (KPF, D). (**B**) Net blue LED light-sensitive current obtained by subtraction of the control ramp from the ramp recorded 3 min after irradiation in the same cell shown in **A**. (**C**,**E**) Averaged time courses (mean ± SEM) of ramp-evoked currents at +80 mV in KFs (n = 9) (**D**) or KPFs (n = 6) (**E**) before, during and after the application of 20.6 J/cm^2^ fluence of blue LED light (a,b). Each graph represents the corresponding points chosen for the analysis shown in (**F**). (**F**) Pooled data (mean ± SEM) of ramp current amplitude at +80 mV, recorded before (a) or after 3 min (b) of blue LED light application in KFs (n = 9) or KPFs (n = 6). Statistical analysis: * *p* < 0.05, paired Student’s *t*-test.

Electrophysiological recordings were performed on fibroblast cells isolated from keloid or perilesional tissues. Human keloid fibroblasts showed, on average, *Vm* = −54 ± 3.9 mV, *Cm* = 47.0 ± 8.3 pF, and *Rm* 467.6 ± 144.0 MΩ and perilesional keloid fibroblasts presented *Vm* = −50.9 ± 7.6 mV, *Cm* = 66.0 ± 27.2 pF, and *Rm* = 159.2 ± 68.1 MΩ. The application of blue LED light at a fluence of 20.6 J/cm^2^ increased the amplitude of outward currents evoked by a voltage ramp protocol (from −80 to +80 mV, 800 ms duration) in cultured human keloid fibroblast (Figure 7A). The net current activated by blue LED light, or blue LED light-sensitive current, is an outward current activated at membrane potentials around −50 mV (Figure 7B), consistently with the activation of a voltage-dependent K^+^ current. The increase reached the maximum level after 3 min of light irradiation (Figure 7C) and it is maintained until 10 min. According to K^+^ currents increase, blue LED light induced a membrane hyperpolarization in keloid fibroblasts, from −54.1 ± 3.9 mV before irradiation, to −57.1 ± 3.9 mV as measured 3 min after irradiation (n = 9). The same dose of blue LED light applied on cultured fibroblasts isolated from keloid perilesional tissues did not modify outward currents elicited by ramp protocol (Figure 7D). The percentage of current amplitude at +80 mV measured 5 min after the application of blue LED light was 125.0 ± 7.5% of control in keloid fibroblast cells and 98.4 ± 5.7% in perilesional fibroblasts (Figure 7E). The Figure 7F summarizes the above results showing that blue LED light irradiation significantly increases total outward currents evoked by the ramp in keloid fibroblasts but not in fibroblasts isolated from perilesional tissue.

### 3.6. Modulation of the Redox State of Cytochrome C

**Figure 8:**
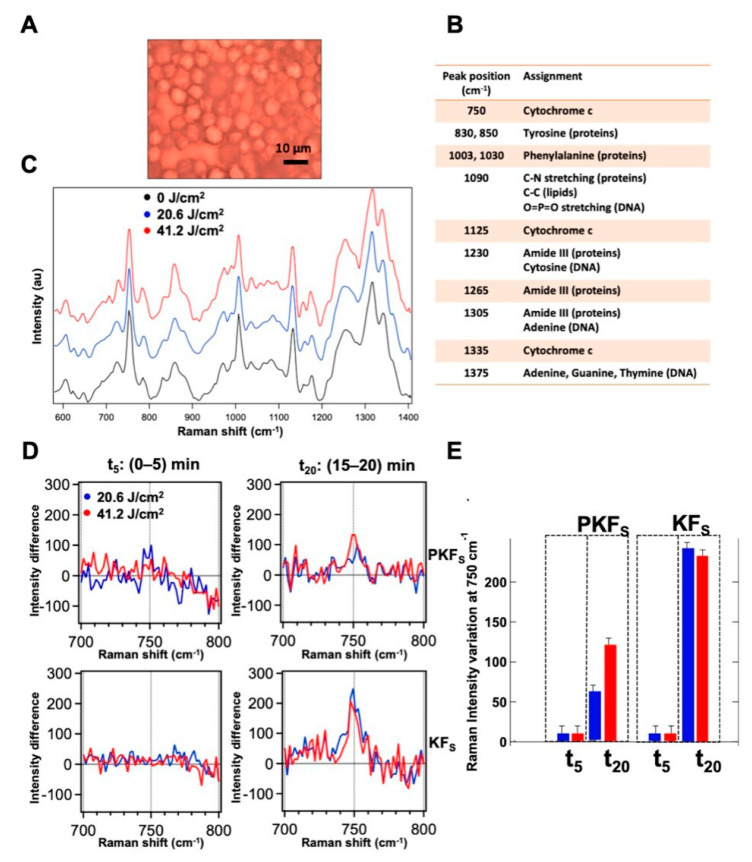
(**A**) Microscopic images of a typical keloid fibroblast pellet prepared for Raman measurements. (**B**) Main assignments of the Raman peaks in keloid fibroblast spectrum. (**C**) Averaged Raman spectra of keloid fibroblasts before (black) and after the application of 20.6 (blue) and 41.2 (red) J/cm^2^ of blue LED light. (**D**) Differential spectra obtained by subtraction of the pre-irradiation spectrum from the post-irradiation spectrum of perilesional and keloid cells averaged in the first 5 min (between 0 and 5 min after irradiation, t5) and in the last 5 min (between 15 and 20 min after irradiation, t20) of the Raman experiment. (**E**) Column bars showing the Raman intensity variation of the peak at 750 cm^−1^ at different times after the irradiation at 20.6 J/cm^2^ (blue) and 41.2 J/cm^2^ (red) of blue LED light.

Cytochrome C was inspected through micro-Raman spectroscopy by performing measurements with 532 nm laser excitation on single cells. The microscopic image of fibroblast cells from keloid in a typical pellet sample prepared for Raman measurements is shown in Figure 8A. Raman spectra of fibroblasts from perilesional and keloid pellets were recorded in three different conditions (before and after the application of 20.6 and 41.2 J/cm^2^ blue light) and the averaged Raman spectrum of the keloid cells at 0, 20.6 and 41.2 J/cm^2^ fluences, respectively, are visualized in Figure 8C. The assignment of the main Raman peaks in the spectra is reported in Figure 7B. We note that the spectral variations in the intensity of the peaks at 750 cm^−1^, 1125 cm^−1^ and 1335 cm^−1^, ascribed to the heme group of Cyt C, have the same sign between the perilesional and keloid fibroblast cells upon blue LED light irradiation. As a consequence, we deduce that the effects on the perilesional and keloid fibroblasts determine an increase in the main Cyt C signals. Through a deeper inspection, these variations appear time-dependent, being more pronounced in the spectra acquired 20 min after the irradiation than in those acquired soon after the blue LED light was turned off. To study this effect, the spectra were averaged within the first 5 min and within the last 5 min over a total time of 20 min of each Raman experiment, for all the samples at the two fluences. Then, a differential Raman analysis was made on the spectra acquired before and after the treatment to quantify the signal variation induced by the irradiation. Figure 8D shows the differential spectra of the cells before the treatment and after the irradiation with 20.6 and 41.2 J/cm^2^ fluences on keloid or perilesional fibroblasts for the peak at 750 cm^−1^. The histogram in Figure 8E shows that in the first 5 min after the irradiation there are no significant changes in the Cyt C Raman peak, both in perilesional and keloid fibroblasts at the two fluences applied. Instead, after 20 min from the treatment, a marked increase in the intensity of 750 cm^−1^ peak can be observed in the all the fibroblasts inspected. This effect is more pronounced in keloid cells; in particular, for the 20.6 J/cm^2^ fluence, the effect on keloid cells is calculated four times higher than the one produced on perilesional cells. For the 41.2 J/cm^2^ fluence, the effect of irradiation after 20 min is higher compared to the 20.6 J/cm^2^ fluence in perilesional cells, while it nearly levels off in keloid cells.

## 4. Discussion

The present work provides an analysis about the effects modulated by a blue LED light (wavelength 410–430 nm, 0.69 W/cm^2^ power density, 3.43–6.87–13.7–20.6–30.9 and 41.2 J/cm^2^ applied doses), performed on cultured fibroblasts isolated from keloids and perilesional tissues. Keloids represent one of the most frequent clinical manifestations of skin fibrosis, together with hypertrophic scars. From a clinical point of view, keloids are described as fibroproliferative dermal noncancerous tumors, however, they can lead to considerable physical and psychological suffering, reducing the quality of life [31]. The scar formation is physiologically involved in the last step of the wound healing process and surely fibroblasts are implicated in the process [32,33]. Currently, no approaches, pharmacological or not, are able to prevent or reduce the recurrence rate of keloids. This paves the way to the employment of alternative therapies, such as PBM [34,35]. Although the mechanism of action of PBM is not fully clarified yet, it is emerging as a key approach in most health-care applications, and blue light has been successfully used in the dermatological and cosmetic fields [36,37]. The lack of validated animal models to study both keloids development and the new possible therapeutic approaches increases the importance of exploiting cell cultures and in vitro investigations, despite their well-known limitations [38]. In our experiments, cell cultures characterization performed by immunocytochemical protocols enabled us to accurately describe isolation and growth of fibroblasts from keloid or perilesional keloid tissues. Through the use of confocal microscopy and specific antibodies, we evidenced a small number of *α*SMA-positive cells in primary cultures obtained both from keloids and perilesional tissues. This marker identifies smooth actin filaments, typical of myofibroblast cells. Our results are consistent with the literature and confirm the role of these cellular phenotype in skin fibrosis [34]. Concerning the cellular and molecular mechanisms responsible for the beneficial therapeutic effects of PBM in wound healing and fibrosis processes, the results from in vitro experiments on different cell types (fibroblasts, osteoblasts, stem cells and lymphocytes) pointed out different hypothesis underlying the PBM mechanism [39,40,41,42,43,44,45,46]. In all these works, and accordingly to Karu’s thesis, [47,48] mitochondria, and in particular Cyt C therein contained, were indicated as one of the most important photoacceptors of visible light and the earliest molecule involved in the trigger of the PBM [49,50]. This process starts with the absorption of specific wavelengths of light by components of the mitochondrial respiratory chain. This primary event generates a signal transmitted at the intracellular level, where it activates a series of active molecules [51]. Among these, the main cited are: cytokines, nitrogen monoxide [40,41] and growth factors; [52,53,54] subsequently, several cellular processes are stimulated, such as the up-regulation of ATP synthesis [55,56] and the cellular metabolism and proliferation increase [50,55,57]. In our work, we conducted a Raman spectroscopy investigation on Cyt C directly measured on single fibroblasts isolated from keloid or from perilesional tissue. According to the literature [57,58], our results indicate that the blue LED light promptly affects the Cyt C causing a change in the redox state of this molecule that can be revealed by a variation in the intensity of specific signals of the Raman spectrum, associated to the heme function of Cyt C. Specifically, an increase in the reduced vs. oxidized species of Cyt C molecule was estimated through Raman experiments upon irradiation of the cells with the blue light [14]. This effect appears time-dependent, showing a precise time pattern: it is immediately activated, and it is more evident after 20 min. This event suggests an amplification of the signal after the initial light stimulus, typical of an ongoing cellular response. Several studies are conducted about the PBM effects of blue light on dermal human fibroblasts, investigating a large number of cellular aspects in response to the irradiation. It has been proved that 60 J/cm^2^ or 90 J/cm^2^ dose of blue light (410–420 nm) significantly reduces fibroblasts viability, while the daily application of 5 J/cm^2^ or 10 J/cm^2^ dose inhibits cell proliferation, partially by inducing oxidative stress [59]. Despite this, other authors reported that the irradiation performed using blue light (470 nm) ranging from 3 J/cm^2^ to 55 J/cm^2^ did not affect cell viability [60]. We tested six doses of blue LED light (3.43–6.87–13.7–20.6–30.9 and 41.2 J/cm^2^) applied directly on keloid-derived fibroblasts and in fibroblasts isolated from perilesional tissues. Our results demonstrate that the blue LED light can modulate both cell metabolism and proliferation, after 24 h only, in a dose-dependent manner, in either type of fibroblasts; after 48 h this effect is still ongoing and appears accentuated. Since some authors have reported the cytotoxic impact of the blue light on cellular viability [61], we performed a trypan blue exclusion test on fibroblast cultures after 24 and 48 h from the application of the maximum dose (41.2 J/cm^2^). Our results did not show colored cells, a clue of cellular death, both in fibroblasts isolated from keloids and from perilesional tissues, at either time tested. Aiming to explore this evidence, we evaluated the number of cells before and after the treatment with blue LED light. It has emerged that the number of cells in controls and treated samples did not change 24 h after the irradiation in keloid-derived fibroblasts, while at 48 h the number of treated cells was significantly reduced in comparison to untreated samples. In fibroblasts isolated from perilesional tissues the number of treated cells decreased by almost 50% 24 h after the treatment. This result demonstrates that the reduction observed by applying the fluence of 41.2 J/cm^2^ in WST-8 and SRB assays, should be ascribed to cell death. To investigate the direct effect of the blue LED light on cultured fibroblasts, we performed an electrophysiological analysis by patch-clamp recordings. These experiments demonstrate that the blue LED light is able to increase outward currents only in keloid fibroblasts. Since this effect was coupled to modest and not significant membrane hyperpolarization and on the basis of electrophysiological characteristics, we suppose that the blue LED light sensitive currents are potassium currents. The increase in potassium conductance may sustain the inhibitory effect on cells proliferation according to the literature [62]. In addition, it is well established that in non-excitable cells, such as fibroblasts, the modulation of these currents is strictly dependent on the variation of membrane potential and influences the cell cycle [63] that subsequently can influence the fibroblasts proliferation rate. Further experiments will be necessary to investigate the eventual modulation of proliferation in keloid-derived fibroblasts and in fibroblasts from perilesional tissue. Taken together, all these data suggest that the irradiation with the blue LED light can be a potential treatment for the prevention and reduction of tissue fibrosis, such as hypertrophic scars and keloids, as reported in the literature [64,65,66]. Overall, PBM may be considered an additional therapy in the management of skin fibrosis.

## 5. Conclusions

In this work, we showed the effects of different doses of blue LED light applied on fibroblast cultures isolated from keloids and perilesional tissues. We observed that blue light can modulate both cell metabolism and proliferation in a dose-dependent manner. High fluence values, as 41.2 J/cm^2^, induce a reduction in cell viability, with the effects onset at different times after treatment, when comparing keloid-derived fibroblasts with fibroblasts isolated from perilesional tissues. We thus focused the study on a lower light dose, and in particular on the 20.6 J/cm^2^ dose effects. The Raman analysis on Cyt C demonstrated the responsiveness of this molecule to the blue light excitation, confirming its role in PBM, and a different behavior in keloid cells. Electrophysiological recordings evidenced an increase in the outward currents only in fibroblasts isolated from keloid tissues. In conclusion, this work supports the hypothesis that PBM can be performed at wavelengths in the blue range of the light spectrum. The effects are different, depending on dose and cell types. PBM can thus be proposed as a promising therapy to be used in the management of cutaneous fibrosis, likely in combination with pre-existing treatments. The different responses given by different fibroblasts to the same dose of light opens the possibility of targeted therapies in different areas of the wound and in wounds with different etiology.

## Figures and Tables

**Table 1:** Doses of blue light used to perform the experiments.

Time (s)	Fluence (J/cm^2^)
5	3.43
10	6.87
20	13.7
30	20.6
45	30.9
60	41.2

**Table 2:** Characteristics of keloid and perilesional tissue used to prepare primary cultures of human fibroblasts.

Fitzpatrick Score	Year of Birth/Sex	Anatomical Site/Size (cm)	Surgery
IV	2004/m	hand/8x4 and perilesional tissue	surgical removal
II	1972/m	helix/1.5 × 1	cryoexcision
II	1975/f	earlobe/1 × 1	cryoexcision
II	1987/m	gluteus/6 × 7 and perilesional tissue	surgical removal
II	1987/m	scapula/5 × 5 and perilesional tissue	surgical removal
V	1980/f	auricle/4 × 2 and perilesional tissue	cryoexcision
V	1980/m	helix/12 × 7	cryoexcision
VI	1989/m	nape/2 × 3.5 and perilesional tissue	cryoexcision
V	1999/m	mandibular/5 × 1.5 and perilesional tissue	cryoexcision
V	1982/m	mandibular/2.5 × 2.5 and perilesional tissue	cryoexcision
V	1977/m	mandibular/8 × 3 and perilesional tissue	cryoexcision

**Table 3:** Pooled data of cell viability in keloid and perilesional fibroblasts (mean ± SD) in control and treated cell cultures, and the relative *p*-values resulting from the unpaired *t*-test.

Sample	Control	Treated J/cm^2^	*p* Value
Keloid 24 h	36.55 (17.91)	37.37 (10.42)	0.761
Keloid 48 h	55.83 (17.39)	40.40 (15.56)	<0.001
Perilesional 24 h	53.20 (17.94)	29.62 (11.02)	<0.001
Perilesional 48 h	53.38 (17.57)	24.13 (12.91)	<0.001

## Abbreviations

The following abbreviations are used in this manuscript:

LED	light emitting diode
PBM	Photobiomodulation
Cyt C	Cytochrome C
ATP	adenosine triphosphate
ECM	extracellular matrix
KFs	Keloid fibroblast cells
KPFs	Keloid Perilesional fibroblast cells
DMEM	Dulbecco Modified Eagle Medium
FBS	Fetal Serum Bovine
RT	Room Temperature
PFA	paraformaldehyde
BSA	Bovine Serum Albumin
HSP-47	Heat Shock Protein-47
*α*SMA	Alpha-smooth muscle actin
SFM	serum-free medium
SRB	Sulforhodamine B based assay
CCK-8	Cell Counting Kit-8
DAPI	4′,6-diamidino-2-phenylindole
GTP	Guanosine triphosphate
EGTA	ethylene glycol tetraacetic acid
HEPES	4-(2-hydroxyethyl)-1-piperazineethanesulfonic acid
EK	potassium equilibrium potential
ECl	chlorine equilibrium potential
